# Glycosaminoglycan from *Ostrea rivularis* attenuates hyperlipidemia and regulates gut microbiota in high‐cholesterol diet‐fed zebrafish

**DOI:** 10.1002/fsn3.2492

**Published:** 2021-07-26

**Authors:** Yan Kong, Ying Li, Zi‐Ru Dai, Mei Qin, He‐Liang Fan, Jun‐Guang Hao, Chen‐Xiao Zhang, Qiu‐Ping Zhong, Cen Qi, Pei Wang

**Affiliations:** ^1^ Guangxi Key Laboratory of Beibu Gulf Marine Biodiversity Conservation Beibu Gulf University Qinzhou China; ^2^ College of Light Industry and Food Engineering Guangxi University Nanning China; ^3^ Qinzhou Key Laboratory of Food Flavor Analysis and Control Beibu Gulf University Qinzhou China

**Keywords:** glycosaminoglycan, gut microbiota, hypolipidemic, *Ostrea rivularis*

## Abstract

Hyperlipidemia an immense group of acquired or genetic metabolic disorders that is characterized by an excess of lipids in the bloodstream. Altogether, they have a high prevalence worldwide and constitute a major threat to human health. Glycosaminoglycans (GAG) are natural biomolecules that have hypolipidemic activity. The purpose of this study was to investigate the potential hypolipidemic effect of glycosaminoglycans extracted from *Ostrea rivularis* (OGAG) on hyperlipidemic zebrafish, as well as the possible underlying mechanism of such effect. Dietary supplementation with OGAG during 4 weeks significantly reduced the serum and hepatic lipid levels and the hepatosomatic index in hyperlipidemic zebrafish. In addition, histopathological showed that OGAG supplementation decreases the volume and number of lipid droplets in hepatocytes. Transcriptome and real‐time quantitative polymerase chain reaction analysis revealed that the gene expression levels of PPARγ, SCD, HMGRA, ACAT2, HMGCS, and HMGCR were significantly downregulated by OGAG treatment in hepatocytes, whereas those of CD36, FABP2, FABP6, ABCG5, and CYP7A1 were significantly upregulated. This suggests that the hypolipidemic effect of OGAG relies on increasing the ketogenic metabolism of fatty acids, inhibiting cholesterol synthesis, and enhancing the transformation of cholesterol to bile acid. Furthermore, OGAG treatment improved gut microbiota imbalance by reducing the *Firmicutes*‐to‐*Bacteroidetes* ratio, increasing the relative abundance of beneficial bacteria (*Bacteroidetes*, *Verrucomicrobia*, *Acidobacteria*, and *Sphingomonas*), and reducing the relative abundance of harmful bacteria (*Proteobacteria*, *Cohaesibacter*, *Vibrio*, and *Terrisporobacter*). These findings highlight the potential benefit of implementing OGAG as a dietary supplement to prevent and treat hyperlipidemia.

## Graphical abstract

Dietary supplementation with OGAG during 4 weeks significantly reduced the serum and hepatic lipid levels and the hepatosomatic index in hyperlipidemic zebrafish.The hypolipidemic effect of OGAG relies on increasing the ketogenic metabolism of fatty acids, inhibiting cholesterol synthesis, and enhancing the transformation of cholesterol to bile acid.OGAG treatment improved gut microbiota imbalance by reducing the *Firmicutes*‐to‐*Bacteroidetes* ratio, increasing the relative abundance of beneficial bacteria (*Bacteroidetes*, *Verrucomicrobia*, *Acidobacteria*, and *Sphingomonas*), and reducing the relative abundance of harmful bacteria (*Proteobacteria*, *Cohaesibacter*, *Vibrio*, and *Terrisporobacter*).

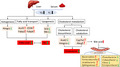

**Abbreviations**
CETP: Cholesterol ester transfer protein
Cyp7a1: Cholesterol 7α‐hydroxylase gene
FASN: Fatty acid synthase
HMGCS: HMG‐coenzyme A (CoA) synthase
IL10: Interleukin 10
LDLRA: Low‐density lipoprotein receptor a
PPARγ: Peroxisome proliferator‐activated receptor γ
SCD: Stearoyl‐CoA desaturase
SREBP1: Sterol regulatory element binding protein‐1c
TNF‐α: tumor necrosis factor α

## 1 INTRODUCTION

Hyperlipidemia is a common lipid metabolism disorder consisting of abnormally elevated levels of any lipid in blood. Hyperlipidemia can cause atherosclerosis and subsequently lead to stroke, hypertension, myocardial infarction, and coronary heart disease, and therefore, poses a serious threat for human health (Farzadfar et al., 2011). Currently, the only available treatment are hypolipidemic drugs such as statins, fibrates, niacin, ezetimibe, and bile acid sequestrants (Taghizadeh et al., 2018). However, these compounds can also have adverse effects with a profound impact on patients' health, such as hepatotoxicity, gastrointestinal dysfunction, myositis, rhabdomyolysis, or renal function damage (Abd & Jacobson, 2011; Abourbih et al., 2009; Sathasivam, 2012). In addition, hypolipidemic drugs are expensive and, hence, place a significant financial burden on the state health system (Yang et al., 2012).

Numerous studies have demonstrated, large amounts of bioactive compounds like polysaccharides extracted from natural resources can ameliorate lipid metabolic disorders due to their high efficacy against hyperlipidemia and their little or none adverse side effects (L. Li & W. Guo et al., 2019; Xie et al., 2016). Crude polysaccharides extracted from abalone viscera reduce lipid levels in plasma and show antiatherogenic activity in a mouse model of a lipid disorder (Liu et al., 2020). The sulfated polysaccharides from *Cytosmear crinite* induced a decrease in low‐density lipoprotein (LDL) and triglycerides (TG) and an increase in high‐density lipoprotein (HDL) blood levels in high‐fat diet rats (Ben Gara et al., 2016). Glycosaminoglycans (GAGs) are long linear heteropolysaccharides widely present in the extracellular matrix of animal tissues (Naderer et al., 2015). These compounds display a variety of biological functions, including antioxidant (Bai et al., 2018), anticoagulant (Guan et al., 2019), hypolipidemic (Liu et al., 2002a), and antitumr activities (Al et al., 2020). Liu et al. (Liu et al., 2002b) recently reported that GAGs extracted from sea cucumber reduce total cholesterol (TC) and LDL cholesterol levels in serum and the atherogenic index in rats fed with a high‐cholesterol diet. Chondroitin sulfate A from *Emerald mussel* enhances the activity of lipoprotein lipase (LPL), leading to a reduction in TC and TG serum levels in patients with hyperlipidemia (Mengjie, 2011). In addition, several studies have demonstrated that natural polysaccharides also exert hypolipidemic effects by modulating the gut microbiota composition (Li, Cheng, et al., 2020; Wan et al., 2020). Sulfate polysaccharide extracted from abalone gonads improves the ecological imbalance of the intestinal flora, inhibits the expression of the GPR43 gene in adipose tissue, and increases GPRA41 expression in mice fed with a high‐fat diet, preventing weight gain and fat accumulation (Liu et al., 2019).

Supplementation with oyster in the diet exhibits a hypolipidemic effect in rats fed with both a normal diet and a high‐cholesterol diet (Iritani et al., 1979). Our objective is to evaluate the potential hypolipidemic effect of glycosaminoglycans from *Ostrea rivularis* (OGAG) and the mechanism underlying such effect. For this purpose, zebrafish were fed with HCD supplemented with OGAG. After 4 weeks, lipid concentrations in serum and liver were analyzed, and histopathological analysis of hepatic tissue was performed. In addition, the expression level of key genes involved in lipid metabolism and inflammation was evaluated with RNA‐seq and RT‐qPCR. Furthermore, the composition of microbiome was assessed through 16S ribosomal DNA sequencing. The results obtained provide theoretical evidence for the further development of OGAG dietary supplements.

## 2 MATERIALS AND METHODS

### 2.1 Materials and chemicals

*Ostrea rivularis* was purchased from a local market in Qinzhou, China, and the OGAG was purified according to the procedure described in previously published literature (Mengjie, 2011). The chemical composition of the purified OGAG is described in Table 1. Reagent kits to quantify TG, TC, LDL‐C, and HDL‐C were purchased from Nanjing Jiancheng Bioengineering Institute. Oil Red O was purchased from Solarbio Biotech Inc. Hematoxylin and eosin (H&E) were purchased from Sigma‐Aldrich. TRIzol reagent was purchased from Invitrogen. The cDNA synthesis kit and TransStart^®^ Top Green qPCR SuperMix were purchased from Beijing TransGen Biotech Inc. Any other reagents used were purchased from Nanning Chemicals and Reagents Co.

**TABLE 1:** Chemical components of OGAG

Composition (%, g/g)	OGAG
Carbohydrates	55.99%
Proteins	26.67%
Uronic acids	0.31%
Acidophobe	1.24%
Glycosaminoglycan	49.05%
Galactosamine	10.61%
Hexosamine	23.50%

### 2.2 Animals and diets

Three‐month‐old wild‐type zebrafish *Danio rerio* were maintained in a circulating water system with the following parameters: a photoperiod consisting of 14 hr light/10 hr dark each day, a temperature of 28 ± 1°C, oxygen saturation greater than 70%, and pH ranging from 7.2 to 7.6. Zebrafish were randomly divided into six groups comprising 60 animals each, placed in separate tanks (15 fish per tank), and fed with specific diets during the following 4 weeks. The groups are referred as follows, depending on the specific diet they received: (1) control (Con), comprising zebrafish fed with a normal diet; (2) high‐cholesterol diet (HCD), zebrafish fed with a normal diet supplemented with 4% cholesterol; (3) simvastatin‐positive control group (SIM), zebrafish fed with an HCD supplemented with simvastatin 200 mg kg^−1^ day^−1^; (4) OGAG‐L, zebrafish fed with an HCD supplemented with a low dose of OGAG (125 mg kg^−1^ day^−1^); (5) OGAG‐M, zebrafish fed with an HCD supplemented with a medium dose of OGAG (250 mg kg^−1^ day^−1^); and (6) OGAG‐H, zebrafish fed with an HCD supplemented with a high dose of OGAG (500 mg kg^−1^ day^−1^). Zebrafish were fed with daily ration of 5% of body weight (adjustment weekly). After the completion of the 4 week treatment period, blood samples from the tail vein of the zebrafish, after fasting for 12 hr, were collected. Hepatic tissue samples were rapidly collected and they were fixed using 4% paraformaldehyde.

### 2.3 Biochemical analysis

Blood samples were collected and incubated at 37°C for 1.5 hr. Samples were centrifuged at 2,500 rpm for 10 min at 4°C to separate the serum from the globular package. TG, TC, LDL‐C, and HDL‐C concentration in the serum was measured using the commercial assay kits from Nanjing Jiancheng Technology Co. based on the manufacturer's instructions.

### 2.4 Histological analysis and oil red O staining

Liver tissue samples that have been previously frozen in liquid nitrogen were sliced at a thickness of 5 µm using a Cryostat (Leica). Sample slices were stained with H&E and Oil Red O as described by Ting et al. (Ting et al., 2018) and Kirsty et al. (Kirsty et al., 2018). The stained sections were observed under the microscope (AxioCam ERc 5s, Carl Zeiss Microscopy) and images were captured at 400x magnifications. Images were analyzed with ImageJ software 8.0.

### 2.5 RNA extraction and quantitative real‐time PCR (RT‐qPCR)

The total RNA was extracted from the liver samples using TRIzol reagent (Invitrogen). The quality of the isolated RNA was assessed using a nucleic acid quantitative device (Qubit). cDNA was synthesized using the cDNA Synthesis Kit (Beijing TransGen Biotech Inc). Relative gene expression levels were determined by quantitative real‐time PCR using the SYBR Green RT‐qPCR kit (Beijing TransGen Biotech Inc) on a QuantStudio 3 Real‐time PCR System. The reaction conditions were as follows: predenaturation at 94°C for 30 s, denaturation at 94°C for 5 s, annealing at 55°C for 15 s, extension at 72°C for 10 s, and the operation was carried out for a total of 35 cycles. Primers used are listed in Table S1. Data were analyzed using the ^ΔΔCt^ method. Expression levels were normalized to the expression of GAPDH gene (Liao et al., 2018; Zhao et al., 2018).

### 2.6 Transcriptome analysis

The cDNA library was constructed and sequenced on Illumina Hisses 2,500 by NoVo gene. Transcriptome sequencing data were analyzed according to the method described by Xie et al. (Xie et al., 2019).

### 2.7 16s rRNA sequencing and analysis

DNA from intestinal samples was extracted following the cetyltrimethylammonium bromide method as previously described elsewhere (Ausubel et al., 1994). The TruSeq^®^ DNA PCR‐Free Sample Preparation Kit was used for library construction. The quality of the library was quantified using the Qubit device and qPCR, and sequenced using a NovaSeq6000 system. Bioinformatic analysis was performed as previously described (Li, Cheng, et al., 2020; Li, Cheng, et al., 2020; L. Li & W. L. Guo et al., 2019).

### 2.8 Statistical analysis

The experimental data are expressed as the mean ± standard deviation (*SD*). Three technical replicates were performed for each assay. Differences among groups were calculated using one‐way analysis of variance (ANOVA) and the LSD test analysis using SPSS 23.0 software (SPSS). Differences between groups were considered statistically significant at *p* < .05. In the figures, different letters indicate significant difference.

## 3 RESULTS

### 3.1 Effects of different OGAG supplementation on growth parameters

**FIGURE 1:**
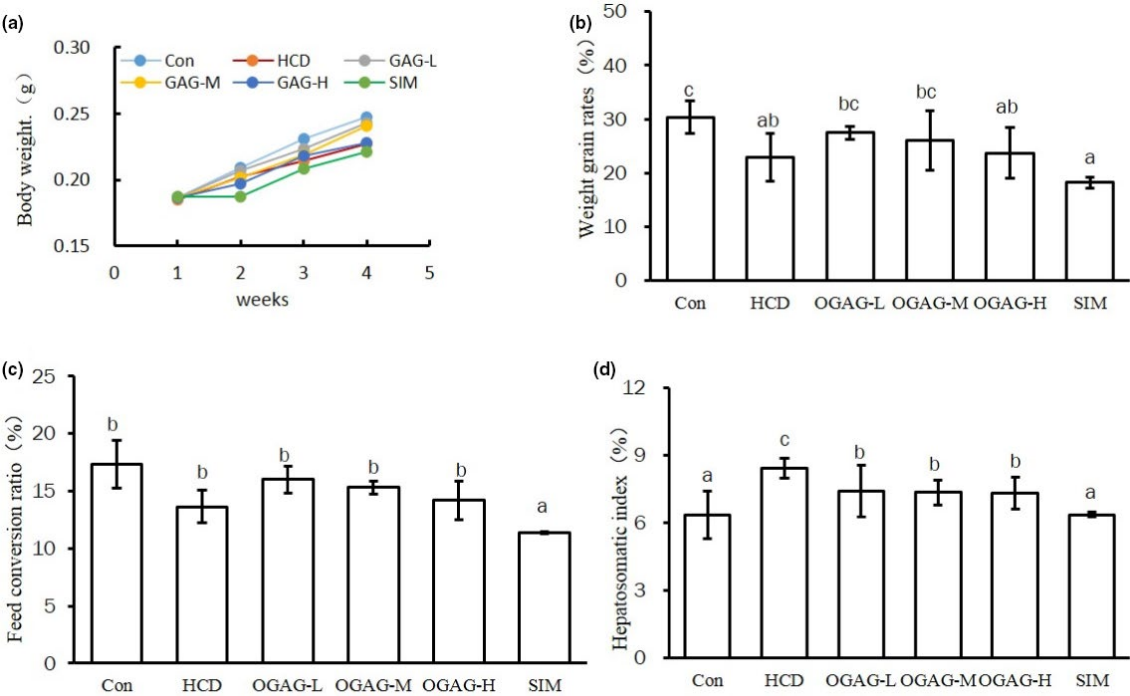
Effects of OGAG supplementation on growth parameters in zebrafish fed with a high‐fat diet for 4 weeks. (a) Relative body weight curves, (b) weight gain rate ((Final Weight‐Initial Weight)/Initial Weight × 100), (c) feed conversion rate was calculated by the formula (body weight gain/total feed intake × 100), and (d) hepatosomatic index was calculated by the formula (liver weight/body weight  × 100). Data are expressed as the mean ± *SD* (*n* = 15). Different letters (superscripts) represent the statistical difference between different groups (*p* < .05)

To analyses the potential hyperlipidemic effect of OGAG, zebrafish were fed during 4 weeks with a high‐cholesterol diet (HCD), or an HCD supplemented with low, medium, or high doses of OGAG (referred to as OGAG‐L, OGAG‐M, and OGAG‐H, respectively). As a negative control, zebrafish were fed with a standard diet (Con); and as a positive control, zebrafish were fed with an HCD supplemented with the lipid‐lowering medication simvastatin (referred as SIM). The HCD, SIM, and OGAG‐H groups showed a decreased weight gain rate in comparison with the control group (Figure 1a,1b, p < .05). Feed conversion ratio (FCR) was calculated as the percentage of weight gain in relation to total feed intake. No significant differences were observed in the FCR, except for the SIM group, which showed a decreased FCR in comparison to the control group (*p* < .05; Figure 1c). In addition, the hepatosomatic index (HSI) was also used as an indicator of nutritional status in fish. HSI was calculated as the percentage of liver weight compared to total body weight. The HCD‐fed group showed a significantly higher HSI compared to the control group as a result of the build‐up of lipids in the liver due to the high‐fat diet. However, the HCD‐induced HSI increase was abolished by supplementation with OGAG, as all groups that received OGAG supplementation in the diet showed HSI lower than the HCD group (*p* < .05; Figure 1d). In addition, supplementation with the lipid‐lowering drug simvastatin also abolished HCD‐induced HSI increase. Altogether, these results indicate that OGAG dietary treatment reduces fat accumulation in the liver of HCD‐fed zebrafish.

### 3.2 Effects of different OGAG supplementation on serum biochemistry

**FIGURE 2:**
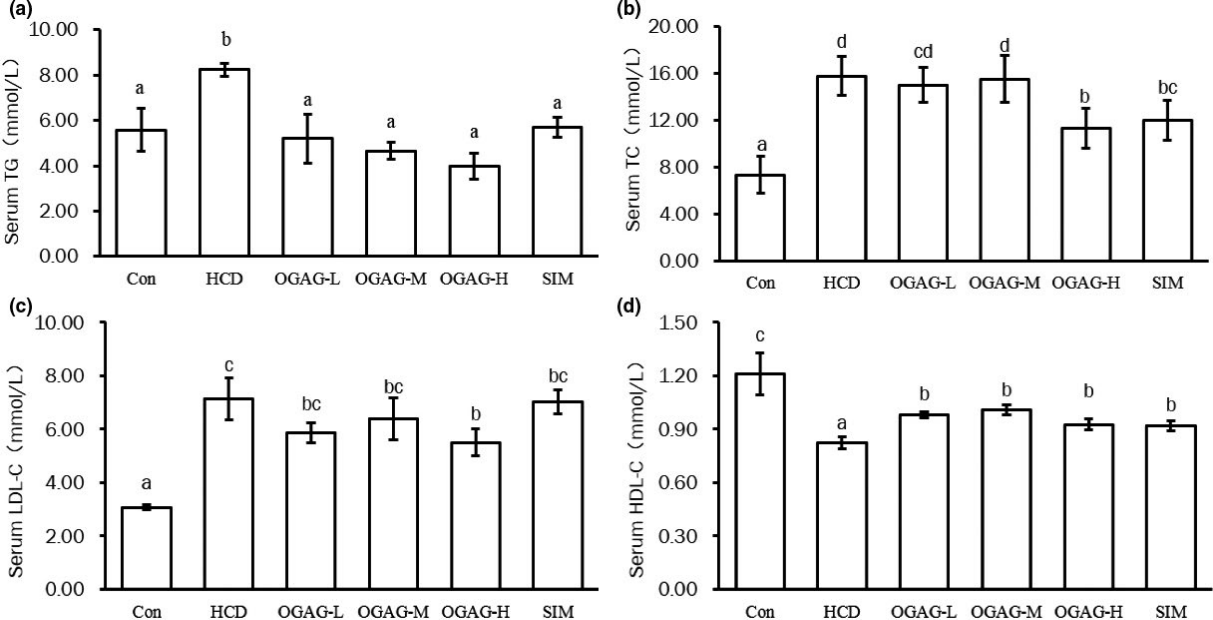
Effects of OGAG supplementation on serum lipid levels in zebrafish fed with high‐fat diets for 4 weeks. (a) Serum triglyceride concentration, (b) total cholesterol serum concentration, (c) low‐density lipoprotein cholesterol serum concentration, and (d) high‐density lipoprotein cholesterol serum concentration. Data are expressed as the mean ± *SD* (*n* = 15). Different letters (superscripts) represent the statistical difference between different groups (*p* < .05)

To evaluate the effect of OGAG supplementation on lipid metabolism, the concentration of TG, TC, HDL‐C, and LDL‐C in serum was determined after the 4 week treatment. HCD led to an increase in TG, TC, and LDL‐C and a reduction in HDL‐C compared to the control group (Figure 2). Supplementation with OGAG tended to prevent these alterations. OGAG effects were especially prominent in the case of the high dose of OGAG, which induced a significant reduction in TG, TC, and LDL‐C levels in comparison to the HCD group (Figure 2, *p* < .05).

### 3.3 Effects of OGAG‐H on hepatic lipid accumulation in HCD‐fed zebrafish

**FIGURE 3:**
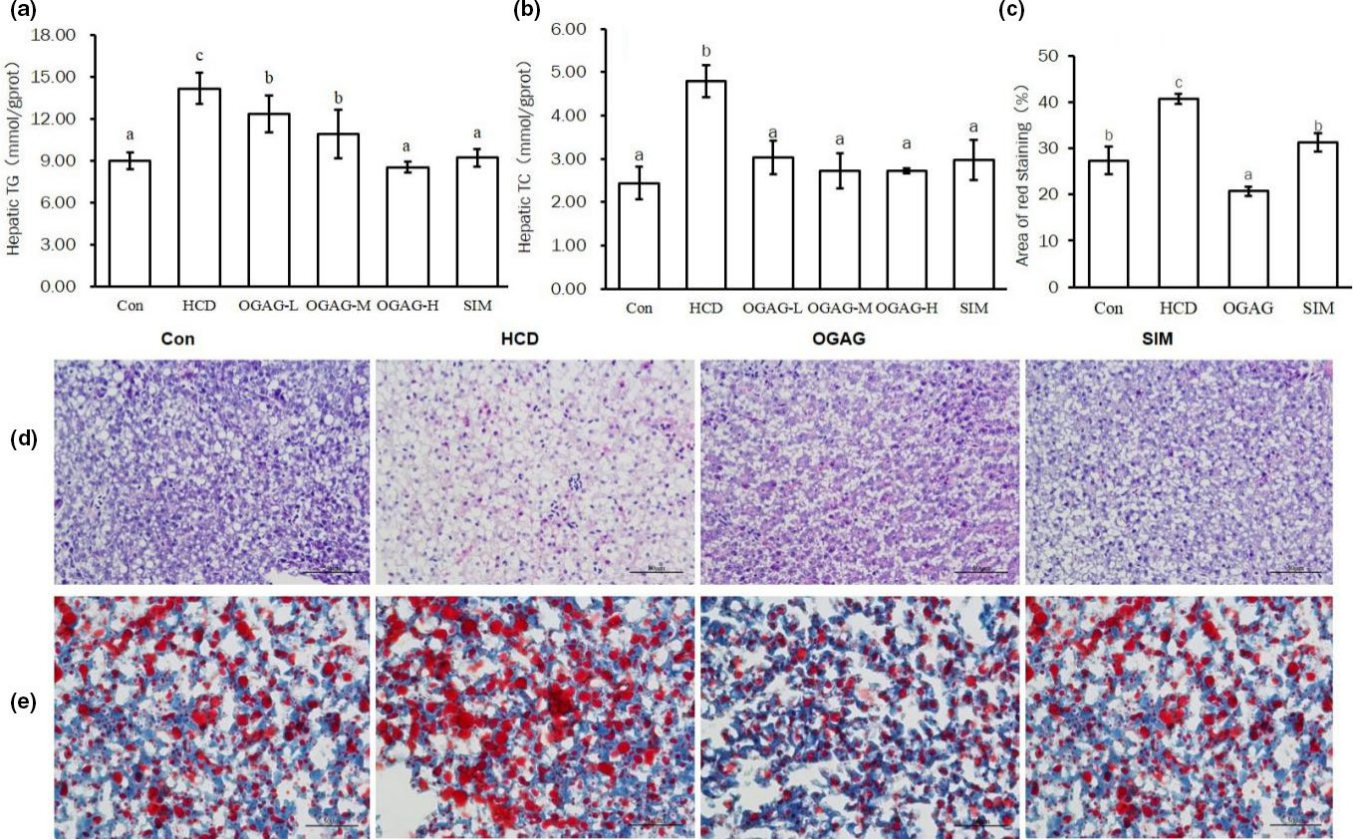
Effects of OGAG‐H on hepatic lipid accumulation in HCD‐fed zebrafish. (a) Hepatic triglyceride hepatic concentration and (b) total cholesterol hepatic concentration. Data are expressed as the mean ± *SD* (*n* = 15). (c) Percentage of Oil Red O stained area. (d) Histopathological analysis of the hepatic tissue sections from zebrafish fed the high‐fat diets for 4 weeks by using H&E staining using 400x magnification, (e) Oil Red O staining of hepatic tissue using 400x magnification. Data are expressed as the mean ± *SD* (*n* = 3). Different letters (superscripts) represent the statistical difference between different groups (*p* < .05)

To further investigate the effects of OGAG supplementation on hepatic lipid content, we analyzed hepatic TC and TG levels. Both TC and TG concentrations were significantly increased in the HCD group. Supplementation with a high dose of OGAG prevented this increase to the same extent as the treatment with simvastatin (Figure 3a,3b). To further analyses the potential benefits of OGAG, histological analysis of liver tissue was performed using H&E and Oil Red O staining. H&E staining shows hepatocyte morphology and macrophage infiltration, whereas Oil Red O reveals the size and position of lipid droplets in the hepatocytes. HCD induced liver damage and accumulation of numerous lipid droplets, but these changes were prevented when high doses of OGAG (500 mg kg^−1^ day^−1^) were administered (Figure 3c,d,e). Analysis of the lipid droplet size quantifying the area stained with Oil Red O showed that OGAG supplementation reduced the lipid droplet size to a greater extent (49%) than simvastatin (23%). Both effects were statistically significant (*p* < .05; Figure 3c). These results demonstrate OGAG‐H supplementation efficiently protects against HCD‐induced hepatic steatosis.

### 3.4 Effects of OGAG supplementation on hepatic lipid metabolism

**FIGURE 4:**
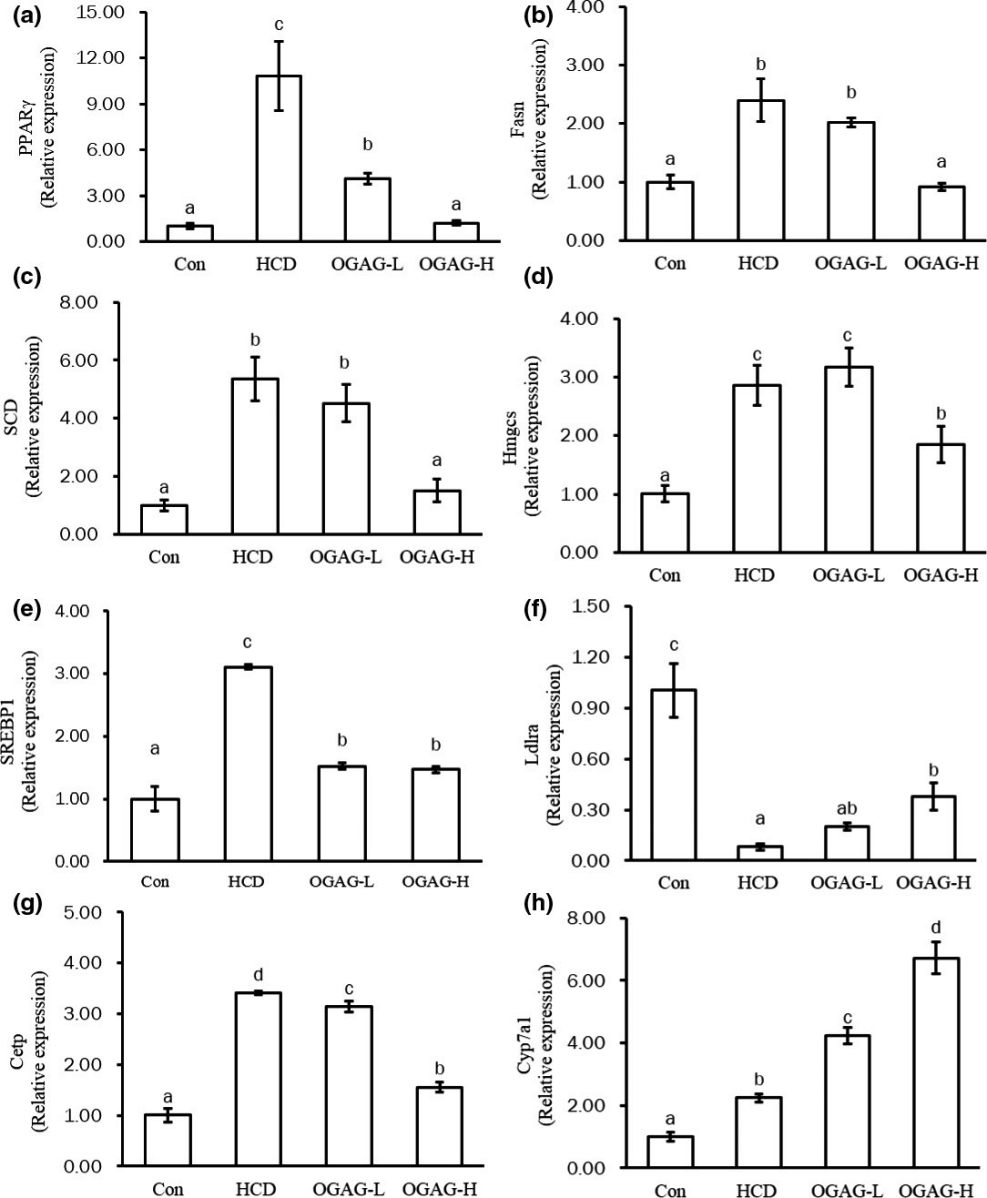
Effects of OGAG supplementation on the hepatic expression levels of genes related to lipid metabolism. (a) PPARγ, (b) FASN, (c) SCD, (d) HMGCS, (e) SREBP1, (f) CETP, (g) LDLRA, and (h) CYP7A1 in the hepatic tissue of high‐fat diet‐fed zebrafish by using RT‐qPCR. Data are expressed as the mean ± *SD* (*n* = 15). Different letters (superscripts) represent the statistical difference between different groups (*p* < .05). Technical triplicates were performed three times

To determine whether the above‐mentioned changes in biochemical parameters were accompanied by changes in gene expression, comparative transcriptome analysis of liver tissues from the control, HCD, and OGAG groups was performed. Several transcripts encoding key molecules involved in lipid metabolism exhibited significantly differential expression between the HCD and OGAG groups based on the transcriptome analysis with RNA‐Seq (Table 2). Eight of these key genes (PPARg/PPARγ,FASN, SCD, HMGS, SREBP1, CETP, LDLRA, and CYP7A1) were selected as representative genes, and their expression levels were evaluated with RT‐qPCR analysis to confirm the expression patterns determined by RNA sequencing (Figure 4).

**TABLE 2:** Genes associated with lipid metabolism that were differentially expressed between the HCD and OGAG groups in hepatic tissue using RNA‐seq

Gene ID	Gene Name	Full Name	Log_2_ Fold Change
ENSDARG00000075931	ACSL5	Acyl‐CoA synthetase long‐chain family member 5	9.54
ENSDARG00000044566	FABP6	Fatty acid binding protein 6	7.94
ENSDARG00000032639	CD36	CD36 molecule (thrombospondin receptor)	7.78
ENSDARG00000006427	FABP2	Fatty acid binding protein 2	5.30
ENSDARG00000069018	CYP7A1	Cytochrome P450, family 7, subfamily A, and polypeptide 1	4.60
ENSDARG00000063078	ABCG5	ATP‐binding cassette, subfamily G (WHITE), and member 5	1.25
ENSDARG00000038618	CPT2	Carnitine palmitoyltransferase 2	−1.24
ENSDARG00000052734	HMGCRA	3‐hydroxy−3‐methylglutaryl‐CoA reductase a	−1.36
ENSDARG00000033662	SCD	Stearoyl‐CoA desaturase	−1.45
ENSDARG00000029476	LDLRA	Low‐density lipoprotein receptor a	−2.01
ENSDARG00000042332	PLIN2	Perilipin 2	−2.08
ENSDARG00000053068	CYP8B1	Cytochrome P450, family 8, subfamily B, and polypeptide 1	−2.11
ENSDARG00000026759	LDLRB	Low‐density lipoprotein receptor b	−2.13
ENSDARG00000031848	PPARG	Peroxisome proliferator‐activated receptor gamma	−2.15
ENSDARG00000053215	ME1	Malic enzyme 1	−2.41
ENSDARG00000104495	PLTP	Phospholipid transfer protein	−2.56
ENSDARG00000103025	HMGCS1	3‐hydroxy−3‐methylglutaryl‐CoA synthase 1	−3.97
ENSDARG00000018923	FAT2	FAT atypical cadherin 2	−4.86
ENSDARG00000007127	ACAT2	Acetyl‐CoA acetyltransferase 2	−6.37

**FIGURE 5:**
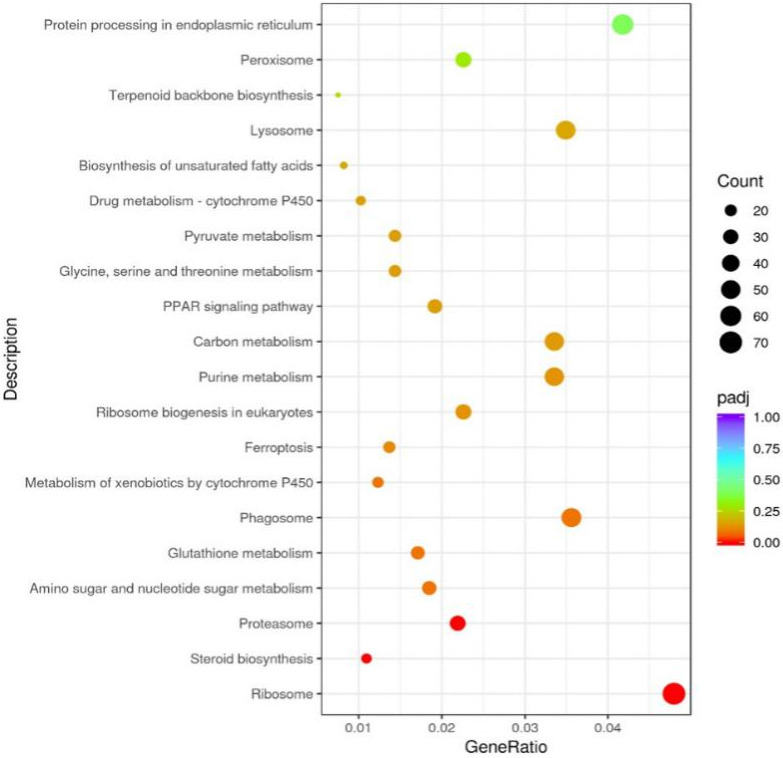
Differentially expressed genes analysis between the HCD and OGAG groups based on KEGG pathway data. Bubble chart shows enrichment of differentially expressed genes in signaling pathways. Y‐axis label represents pathway, and X‐axis label represents Gene Ratio (the number of genes in this entry is a percentage of all genes). Size and color of the bubble represent the amount of differentially expressed enriched in pathway, and the enrichment significance as the rich factor padj, respectively

The main metabolic and signal transduction pathways in which differentially expressed genes participate were analyzed using KEGG enrichment analysis (Figure 5 and Figure S1). Many differentially expressed genes that are relevant to lipid metabolism were enriched by KEGG pathway analysis, such as steroid biosynthesis, proteasome, metabolism of xenobiotics by cytochrome P450, carbon metabolism, PPAR signaling pathway, biosynthesis of unsaturated fatty acids, terpenoid backbone biosynthesis, and peroxisome.

### 3.5 Effects of OGAG supplementation on inflammation in liver

**FIGURE 6:**
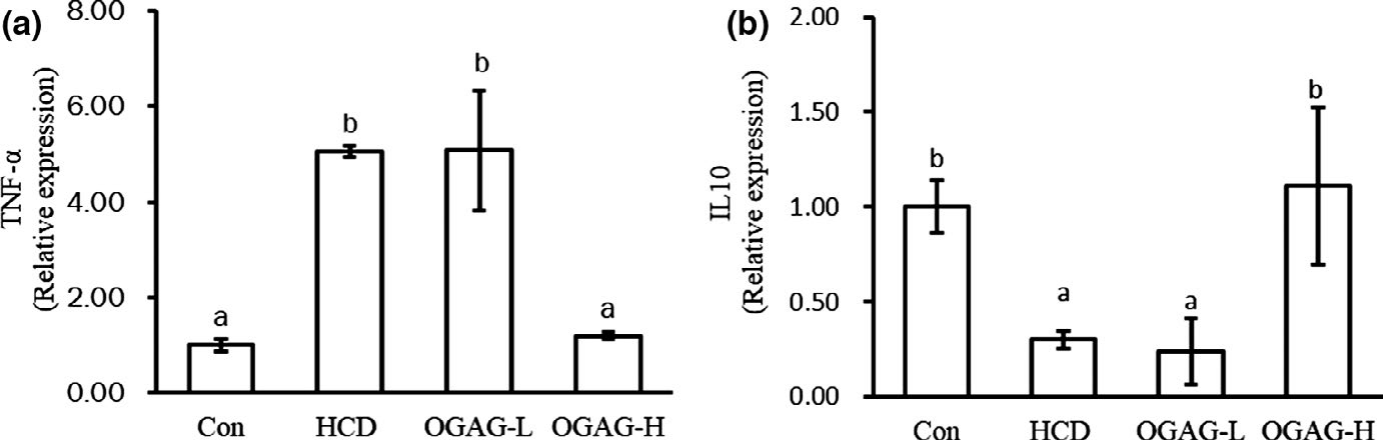
Effect of OGAG treatment on the expression levels of inflammatory cytokines in the hepatic tissue of HC diet‐fed zebrafish using RT‐qPCR. (a) Relative expression levels of TNF‐α and (b) relative expression levels of IL‐10. Data are expressed as the mean ± *SD* (*n* = 15). Different letters (superscripts) represent the statistical difference between different groups (*p* < .05). Technical triplicates were performed three times

In the recent years, several studies have shown that high‐fat diet induces inflammation (Duan et al., 2018). In line with these reports, the proinflammatory factor TNF‐α was significantly upregulated in the HCD group in comparison to the control group, whereas the antiinflammatory cytokine IL‐10 was significantly downregulated (*p* < .05, Figure 6). These changes were abolished with a high‐dose supplementation of OGAG, but not with a low dose (Figure 6). These results indicate that OGAG treatment can alleviate HCD‐induced inflammation in zebrafish.

### 3.6 Effect of OGAG supplementation on the gut microbiota

Emerging research has highlighted the detrimental impact of a high‐fat diet on gut microbiota (Murphy et al., 2015). To investigate the effect of HCD and OGAG supplementation on the zebrafish gut microbiome composition, high‐throughput sequencing of 16S rDNA was performed. Alpha‐diversity estimates including diversity measures (Shannon and Simpson indexes) and richness (ACE/Chao1 ratio) were calculated using the raw count data. The Shannon index in the HCD group was significantly lower than in the other three groups, whereas the Simpson index showed the opposite trend (Table 3). There was no significant difference in the ACE/Chao1 ratio among all treatment groups, which suggests that OGAG‐treated group did not result in an improved richness (Table 3). These results demonstrate that only the gut microbial diversity in the OGAG‐treated group was increased.

**TABLE 3:** Alpha diversity estimates: Shannon and Simpson indexes and ACE/Chao1 ratio. Data are expressed as the mean ± *SD* (*n* = 15). Different letters (superscripts) represent the statistical difference between different groups (*p* < .05)

Group	Shannon	Simpson	ACE/Chao1
Con	0.483 ± 0.079a	0.803 ± 0.022b	1.045 ± 0.008a
HCD	1.318 ± 0.055c	0.197 ± 0.093a	1.039 ± 0.007a
OGAG	0.834 ± 0.095b	0.731 ± 0.209b	1.016 ± 0.027a
SIM	0.949 ± 0.017b	0.663 ± 0.042b	1.006 ± 0.018a

**FIGURE 7:**
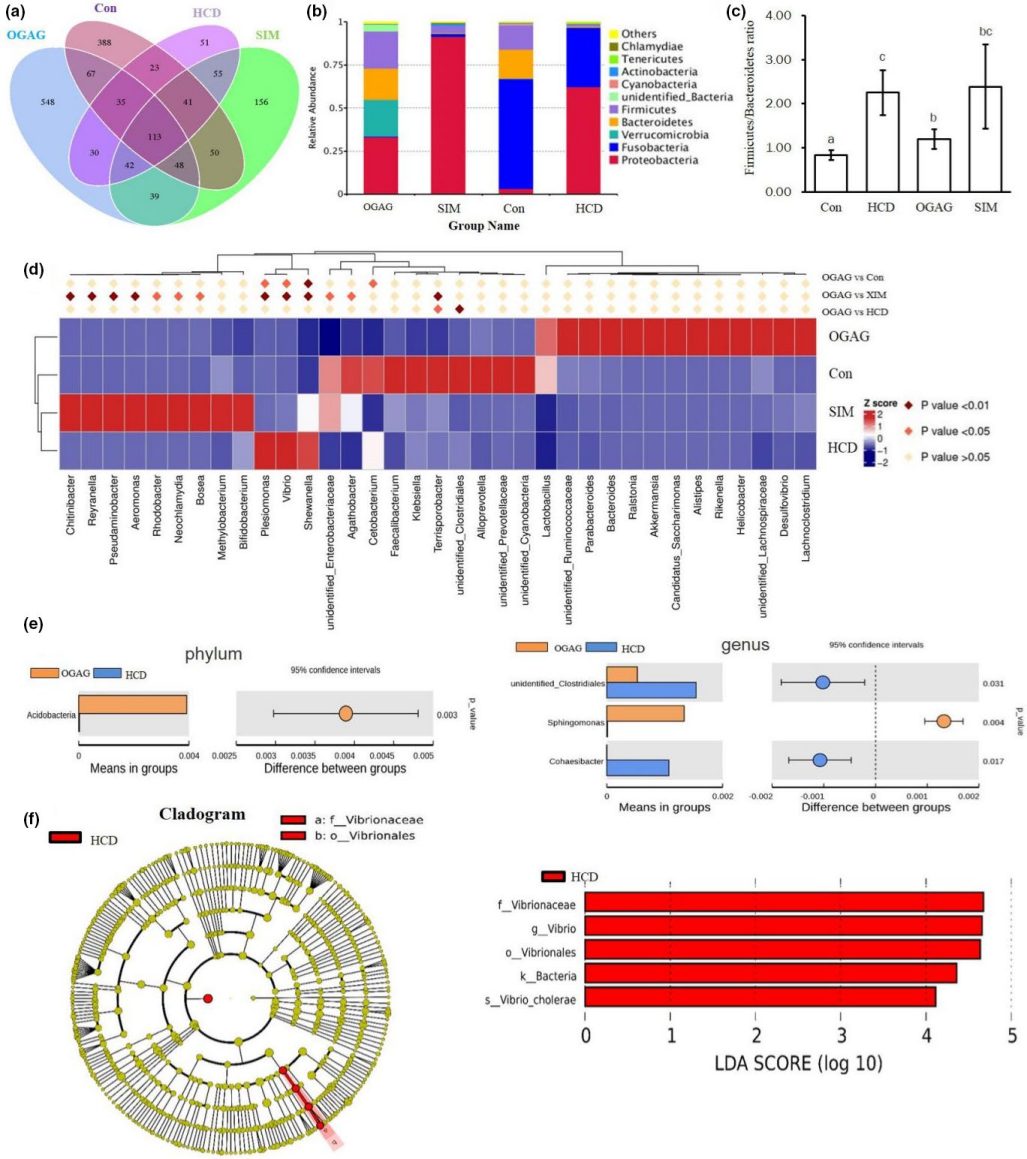
Effects of OGAG treatment on gut microbiota in the zebrafish fed with a high‐fat diets for 4 weeks. (a) Venn diagram of OTUs in the gut microbiota among the four groups, (b) relative abundances of the gut microbiota at the phylum level, (c) *Firmicutes*‐to‐*Bacteroidetes* ratio, (d) heatmap of significant differences in gut microbiota between different treatment groups at the genus level, (e) *T‐*test analysis of species differences between OGAG (orange) and HCD (blue) groups, and (f) cladogram and histogram of LDA effect value based on linear LEfSe. Only LDA score >4 is presented

To assess the differences in microbiome taxa among groups, an in‐depth community analysis was conducted. A total of 84,219, 83,911, 75,526, and 80,333 bacterial 16S rDNA gene valid sequences were obtained from the control, HCD, OGAG, and SIM groups, respectively (Table S2). A Venn diagram was generated to show the shared and unique operational taxonomical units (OTUs) regardless of their relative abundance. The OGAG‐treated group had 388 distinct microbes, whereas the HCD group only had 51 (Figure 7a).

At the phylum level, the relative abundance of both the *Firmicutes* and *Bacteroides* in HCD group was decreased, which was prevented by OGAG treatment (Figure 7b). The *Firmicutes*/*Bacteroidetes* (F/B) ratio of the HCD group was significantly higher than the control group (*p* < .05), which was significantly reduced by OGAG supplementation (*p* < .05; Figure 7c). Additionally, we observed an increase in the relative abundance of *Proteobacteria* in HCD and SIM groups and an increase in relative abundance of *Verrucomicrobia* in the OGAG‐treated group (Figure 7b).

Significant differences between species were also found at the genus level. Supplementation with OGAG led to a significant decrease in the number of *Terrisporobacter* compared with the HCD (*p* < .05) and SIM groups (*p* < .01) and a decrease in unidentified *Clostridiales* in comparison to the HCD group (*p* < .01) (Figure 7d).

Welch's *t* test was used to compare the abundance at the phylum and genus level (Figure 7e) between the HCD and OGAG groups. The relative abundance of *Acidobacteria* at the phylum was significantly increased in the OGAG‐treated group compared to the HCD group. The abundance of *Cohaesibacter* genus and the unidentified *Clostridiales* genus was significantly decreased in the OGAG‐treated group compared to the HCD group, whereas the relative abundance of *Sphingomonas* was greatly increased.

To further identify the distinct bacterial taxa among control, HCD, and OGAG‐treated groups, linear discriminant analysis with effect size (LEfSe) was conducted (Figure 7f). The LEfSe cladogram and the histogram of linear discriminant analysis showed that there were no significant differences in bacterial taxa between the control and OGAG‐treated groups. However, several taxa showed a different proportion in the HCD group, including *Vibrionaceae, Vibrionales, Vibrio*, and *Vibrio cholerae*.

## 4 DISCUSSION

The results presented in this report show that high‐fat diet induces abnormal lipid metabolism and energy metabolism imbalance, features usually present in animal models of hyperlipidemia (Kabir et al., 2020). HCD increased serum and hepatic lipid levels and the HSI, evidencing that high‐fat diet results in lipid accumulation in zebrafish. Supplementation with OGAG reduced the hepatosomatic index, a result in line with previous reports showing the beneficial effects of dietary treatment with natural polysaccharides in animal models of hyperlipidemia (Wang et al., 2020; Zhu et al., 2018). In addition, OGAG treatment improved serum and hepatic lipid parameters. Specifically, OGAG administration significantly reduced TC, TG, and LDL‐C serum levels and increased HDL‐C serum levels; and significantly decreased TC and TG hepatic concentration in hyperlipidemic zebrafish. Furthermore, histopathological analysis showed that supplementation with the high dose of OGAG (500 mg kg^−1^ day^−1^) reduced the lipid accumulation and the size of fat droplets in the liver of hyperlipidemic zebrafish. These results are in line with previous studies reporting that the exopolysaccharides isolated from kefir grains can effectively restrain lipid accumulation (Lim et al., 2017). In addition, after 4 weeks of high cholesterol diet, the weight gain rate of HCD group, SIM group, and OGAG‐H group was significantly lower than that of the control group. Statins have been reported to have toxic side effects on the muscles and liver, such as significantly reduced food intake, weight loss, and liver damage (Yu et al., 2021; Yves et al., 1990). Therefore, it is speculated that the lower weight gain rate of HCD group, SIM group, and OGAG‐H group than CON group may also had been caused by liver damage of zebrafish after cholesterol or SIM treatment or high‐dose OGAG‐H treatment. However, the weight gain rate in OGAG‐L and OGAG‐M groups was similar to that in the CON group, possibly due to the hepatoprotective activity of low‐dose glycolaminoglycan (Song et al., 2017), which can reduce the weight loss induced by high cholesterol diet. However, the specific mechanism of toxicity needs to be further explored.

The liver is an essential organ in the regulation of lipid metabolism, including the metabolism of TG and TC (Huang et al., 2018; Ting et al., 2018; X. Z. Wan et al., 2020a; Zeng et al., 2020). TG represent the major form of lipid storage in the liver. TG break down and produces free fatty acids (Wan et al., 2020), which can be catabolized through β‐oxidation and ketogenesis (La Paglia et al., 2017). In the case of cholesterol, the liver is responsible for both its synthesis and transformation. Cholesterol is synthesized through the mevalonate pathways, a route that starts with Acetyl‐coA and whose rate is controlled by the enzyme HMGCR (Zeng et al., 2020). Later on, cholesterol can be oxidized by the CYP7A1, which is the first and rate‐limiting step in bile acid synthesis (Davis et al., 2002; Fava et al., 2018). To investigate the hypolipidemic mechanism of OGAG in hyperlipidemic zebrafish, liver transcriptome analysis was performed, focusing specifically on mRNA levels of key genes for lipid metabolism. The relative expression of lipogenic genes such as PPARγ, FASN, SREBP‐1, FASN, SCD, and HMGCRA was significantly downregulated by dietary treatment with high dose of OGAG. All these genes play an essential role in lipid biosynthesis and storage: PPARγ and SREBP1 are transcription factors that promote fat synthesis and storage in adipose tissue by positively regulating the expression of key lipogenic genes (Evans et al., 2004), like FASN (J. Li, Fang, et al., 2020); SCD is a key enzyme step in the formation of monounsaturated fatty acids (Brown & Rudel, 2010), HMGCRA is an essential enzyme for the synthesis of cholesterol (Kim et al., 2019). These results indicate that supplementation with OGAG inhibits the expression of key lipogenic genes, thereby reducing lipid accumulation in the liver of hyperlipidemic zebrafish. In addition, OGAG intervention inhibited the expression of CETP and LDLRA. CETP is an enzyme that combines with HDL to reverse transfer of TG to HDL, which accelerates the catabolism of HDL (Shrestha et al., 2018). This may be the main reason for the increase in HDL‐C levels in OGAG treatment group.

OGAG supplementation induced the upregulation of genes related to fatty acids degradation, like CYP7A1, CPT1α, and LDLRA. CYP7A1 catalyzes the oxidation of cholesterol to form bile acid, and CPT1α is the first enzyme in fatty acid β‐oxidation (Moody et al., 2018). LDLRA increases LDL‐c levels (Xie et al., 2019). OGAG treatment promoted the expression of LDLRA in the liver of hyperlipidemic zebrafish, which thereby reduces the content of LDL‐C. Furthermore, molecules implicated in fatty acid transport, like ABCG5, CD36, ACSl5, FABP2, and FABP6, were also upregulated.

**FIGURE 8:**
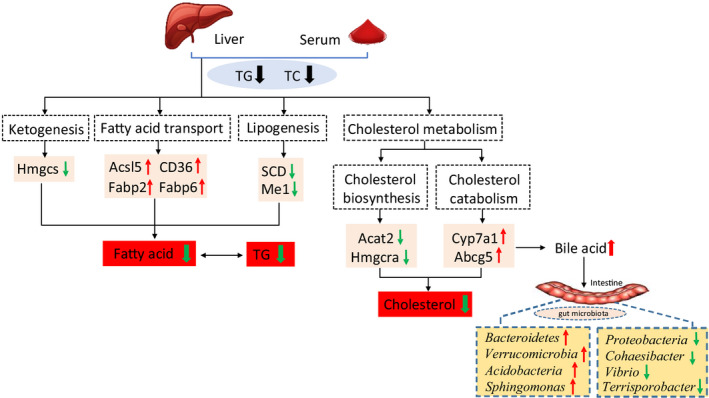
Suggested mechanisms for the attenuation of hyperlipidemia and regulation of gut microbiota by the glycosaminoglycan from *Ostrea rivularis* in zebrafish fed with a high‐cholesterol diet

The above results demonstrate that OGAG exerts its hypolipidemic mechanism regulating the expression levels of key lipogenic and decomposition genes in hepatic tissues. Specifically, OGAG intervention inhibits lipid production, reduces fatty acid accumulation through ketogenic pathways and fatty acid transport pathways, and also promotes the conversion of cholesterol into bile acids (Figure 8).

Polysaccharides can promote the growth of certain intestinal bacteria and change the diversity of gut microbiota (Wan et al., 2020). OGAG treatment increased the relative abundance of *Bacteroidetes*, *Verrucomicrobia*, *Acidobacteria*, and *Sphingomonas*; whereas it reduced the abundance of *Proteobacteria*, *Cohaesibacter*, *Vibrio*, and *Terrisporobacter*. Previous studies have shown that a high‐fat diet can improve the relative abundance of *Firmicutes* and reduces the relative abundance of *Bacteroidetes* (Round & Mazmanian, 2009). The results provided by this study show that HCD induced a decrease in the relative abundance of *Firmicutes*. It is possible that the elevated cholesterol favored *Proteobacteria* proliferation, thereby competitively suppressing the rise of *Bacteroidetes*. OGAG supplementation reduced the *Bacteroides*‐to‐*Firmicutes* ratio, which is in line with a previous study showing that sulfated polysaccharide from sea cucumber can reverse the *Bacteroides*‐to‐*Firmicutes* ratio (Zhu et al., 2018). In addition, OGAG supplementation induced that *Verrucomicrobia* became the unique dominant bacterial population. *Verrucomicrobia and Bacteroidetes* secrete bacterial bile acid sterol dehydrogenase, which further dehydrogenates the decomposed primary bile acid; thus, promoting the catabolism of cholesterol (Chen et al., 2018). OGAG supplementation also leads to a decrease in the relative abundance of *Terrisporobacter,* which is positively correlated with the level of TG (Lee et al., 2020), and therefore, could explain the observed decrease in TG levels. Furthermore, OGAG treatment significantly inhibited the colonization of *Vibrio Cholerae* in the zebrafish intestinal. As *Vibrio cholerae* can cause intestinal infections (Senderovich et al., 2010), it could explain the reduction in inflammatory markers observed. Taken together, these results suggest that OGAG treatment has a beneficial effect on gut microbiota diversity and composition in hyperlipidemic zebrafish, which can provide academic foundation for the hyperlipidemic effect of OGAG.

The present study shows that dietary supplementation with OGAG can significantly improve zebrafish hyperlipidemia symptoms such as hepatosomatic index and blood and serum lipid levels. The underlying hypolipidemic mechanism of OGAG relied on the transcriptional regulation of key genes involved lipid metabolism. In addition, OGAG also improved the diversity and richness of gut microbiome, which could also be associated with the hypolipidemic effect of OGAG, and selectively changed the abundance of gut microbes in hyperlipidemic zebrafish. These findings suggest the potential benefit of dietary treatment with OGAG for hyperlipidemic patients and provide new insights for the exploitation and utilization of OGAG as a potential functional food for prevention and alleviation of lipid accumulation.

## CONFLICT OF INTEREST

The authors declare no competing financial interest.

## AUTHOR CONTRIBUTION

**Yan Kong:** Formal analysis (equal); Resources (equal); Software (equal); Writing‐original draft (equal); Writing‐review & editing (equal). **Ying Li:** Formal analysis (equal). **Ziru Dai** **:** Conceptualization (Equal); Data curation (Equal); Funding acquisition (Equal); Writing‐review & editing (Equal). **Mei Qin:** Methodology (equal). **Heliang Fan:** Validation (equal). **JunGuang Hao:** Conceptualization (equal). **Chenxiao Zhang:** Funding acquisition (equal); Investigation (equal). **QiuPing Zhong** **:** Visualization (Equal). **Cen Qi:** Investigation (equal). **Pei Wang:** Software (equal).

## ETHICAL APPROVAL

This study was approved by the Institutional Review Board of the Animal Care Review Committee, Beibu Gulf University.

## Supporting information

Supplementary Material

## Data Availability

The data that support the findings of this study are available on request from the corresponding author. The data are not publicly available due to privacy or ethical restrictions.
