# Supplementary material for: Glycosaminoglycan from Ostrea rivularis attenuates hyperlipidemia and regulates gut microbiota in high‐cholesterol diet‐fed zebrafish
Source: Food Sci Nutr. 2021 Jul 26;9(9):5198–210. doi: 10.1002/fsn3.2492 (PMC8441474; doi:10.1002/fsn3.2492)
Supplement: Supplementary file 1 — Supplementary Material [file FSN3-9-5198-s001.docx]

**Supplementary information**

Figure S1. Transcriptomic analysis. (A) Volcano plot. The log2 FoldChange indicates the mean expression level for each gene. Each dot represents one gene. After OGAG administration, blue dots represent no significant differentially expressed genes between OGAG group and HCD group, the green dots represent down-regulated genes and red dots represent up-regulated genes. (B) GO classifications of the differentially expressed genes between OGAG group and HCD group. All of the differentially expressed genes were assigned to three categories: cellular component, molecular function, and biological process. Top 10 most enriched GO terms of each category are shown in Figure S1B.


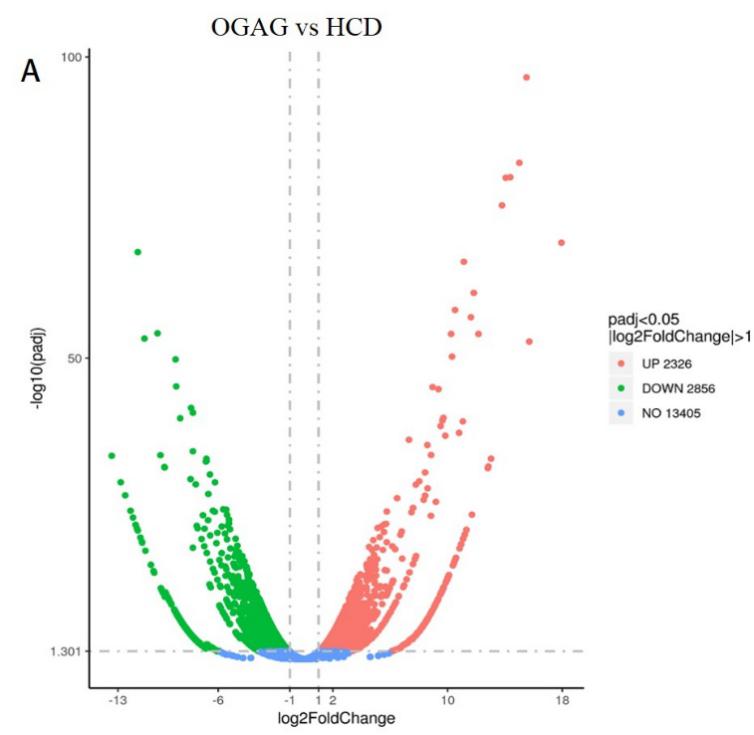


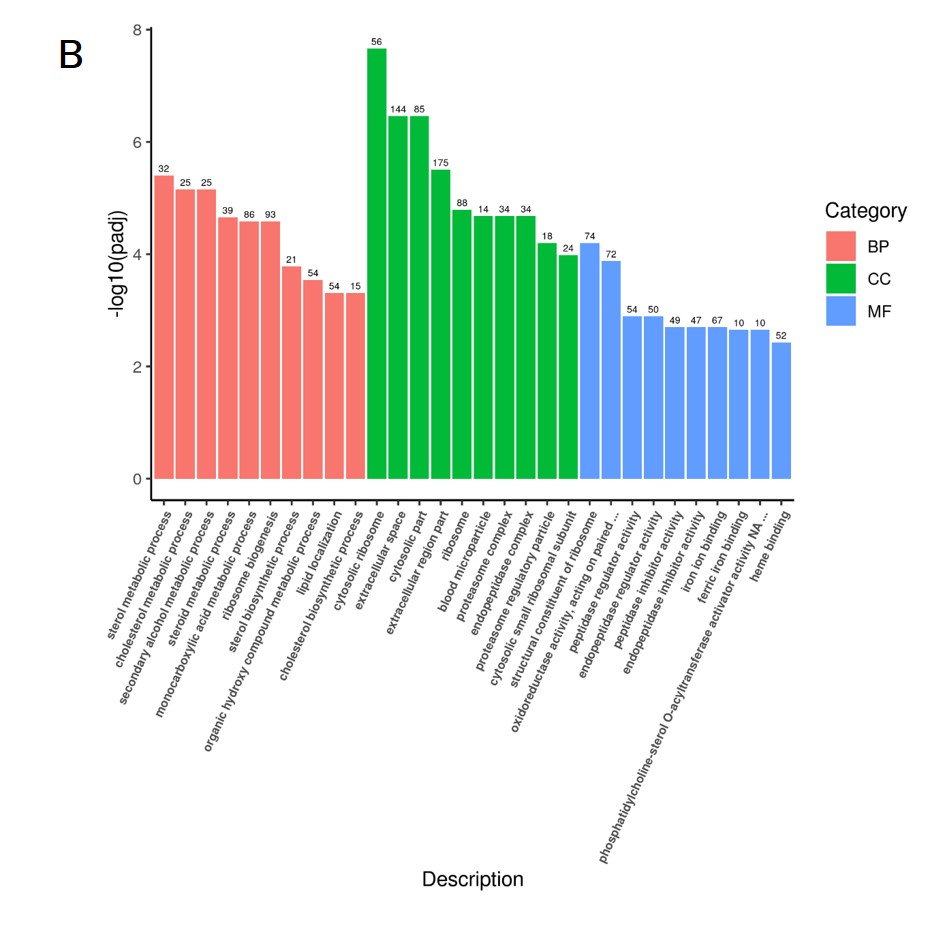


Table S1 Primer sequence of RT-qPCR amplified genes

| Genes | Amplicon length | Primer sequences | |
| --- | --- | --- | --- |
| *GAPDH* | 168 | Forward | 5' TGAGGTTAAGGCAGAAGGCG 3' |
|  |  | Reverse | 5' CCCTTAATGTGAGCAGAAGCC 3' |
| *LDLRA* | 152 | Forward | 5' ATTCACCTGTACCGCCTGAC 3' |
|  |  | Reverse | 5' GGGTTGTCAAAGTGGATGCT 3' |
| *CYP7A1* | 165 | Forward | 5' ACAGTCAAGCTCTACTTCCACC 3' |
|  |  | Reverse | 5' TCTGCAGGATGTCTTCTGCG 3' |
| *SCD* | 191 | Forward | 5' CTCACACTCCTCTGGGCTTTT 3' |
|  |  | Reverse | 5' AAGGCCATGGAGTTTCCGAT 3' |
| *HMGCS* | 96 | Forward | 5' AGGAGTATGATGGAGTCGGG 3' |
|  |  | Reverse | 5' CAGAGAGTTGATGTCCTCG 3' |
| *Fasn* | 250 | Forward | 5' ATCTGTTCCTGTTCGATGGC 3' |
|  |  | Reverse | 5' AGCATATCTCGGCTGACGTT 3' |
| *PPARγ* | 186 | Forward | 5' CACTCTCCGCTGATATGGTGG 3' |
|  |  | Reverse | 5' TTGGGTCATTCTGTGTTGGGT 3' |
| *SREBP1* | 250 | Forward | 5' CATCCACATGGCTCTGAGTG 3' |
|  |  | Reverse | 5' CTCATCCACAAAGAAGCGGT 3' |
| *Cetp* | 210 | Forward | 5' TCCCCCAGTGATCAAATC 3' |
|  |  | Reverse | 5' CCAGAGATACTGCGCACAAA 3' |
| *IL10* | 113 | Forward | 5' TAAAGCACTCCACAACCCCA 3' |
|  |  | Reverse | 5' GACCCCCTTTTCCTTCATCTTTTC 3' |
| *INFα* | 142 | Forward | 5' ATCACCACACCTTCAGCTTCCT 3' |
|  |  | Reverse | 5' GTTGGAATGCCTGATCCACAC 3' |

Table S2 Statistical analysis of biological information of different samples

| Groups | Reads | Goods coverage | Avg len(bp) |
| --- | --- | --- | --- |
| Con | 84219±7317a | 0.9987±0.00a | 405±5a |
| HCD | 83911±2299a | 0.9990±0.00a | 421±9b |
| GAG | 75526±5349a | 0.9980±0.00a | 418±6b |
| SIM | 80333±4919a | 0.9993±0.00a | 421±4b |

Different letters in the same column indicate significant differences at p < 0.05.
